# Experiences of Using a Consumer-Based Mobile Meditation App to Improve Fatigue in Myeloproliferative Patients: Qualitative Study

**DOI:** 10.2196/14292

**Published:** 2019-07-22

**Authors:** Jennifer Huberty, Ryan Eckert, Linda Larkey, Lynda Joeman, Ruben Mesa

**Affiliations:** 1 College of Health Solutions Arizona State University Phoenix, AZ United States; 2 Mays Cancer Center University of Texas Health San Antonio MD Anderson Cancer Center San Antonio, TX United States; 3 College of Nursing and Health Innovation Arizona State University Phoenix, AZ United States; 4 Lynda Joeman Research Consultancy Tonbridge, Kent United Kingdom

**Keywords:** mindfulness, meditation, mobile phone, mHealth, digital health, cancer

## Abstract

**Background:**

Myeloproliferative neoplasm (MPN) patients suffer from long-term symptoms and reduced quality of life. Mindfulness meditation is a complementary therapy shown to be beneficial for alleviating a range of cancer-related symptoms; however, in-person meditation interventions are difficult for cancer patients to attend. Meditation via a mobile phone app represents a novel approach in MPN patients for delivering meditation.

**Objective:**

The study aimed to report MPN patients’ (ie, naïve or nearly naïve meditators) perceptions of meditation and explore their experiences in the context of using a mobile phone for meditation after participation in an 8-week consumer-based meditation app feasibility study.

**Methods:**

MPN patients (n=128) were recruited nationally through organizational partners and social media. Eligible and consented patients were enrolled into 1 of 4 groups, 2 that received varying orders of 2 consumer-based apps (*10% Happier* and *Calm*) and 2 that received one of the apps alone for the second 4 weeks of the 8-week intervention after an educational control condition. Participants were asked to perform 10 min per day of mobile phone-based meditation, irrespective of the app and order in which they received the apps. At the conclusion of the study, participants were asked whether they would like to participate in a 20-min phone interview comprising 9 to 10 questions to discuss their perceptions and experiences while using the mobile phone meditation apps. The interviews were transcribed verbatim and imported into NVivo 12 (QSR International) for coding and analysis, using a combination of deductive and inductive methods to organize the data, generate categories, and develop themes and subthemes.

**Results:**

A total of 48 MPN patients completed postintervention interviews, of which 29% (14/48) of the patients only used the *10% Happier* app, 21% (10/48) of the patients only used the *Calm* app, and 46% (22/48) of the patients used both apps during the 8-week intervention. Themes identified in the analysis of interview data related to (1) perceptions of meditation before, during, and after the study, (2) perceptions of the *Calm* app, (3) perceptions of the *10% Happier* app, (4) perceived impacts of using the meditation apps, (5) overall experiences of participating in the study, (6) recommendations surrounding meditation for other MPN patients, and (7) plans to continue meditation.

**Conclusions:**

The qualitative findings of this study suggest that MPN patients who are naïve or nearly naïve meditators perceived mobile phone meditation as enjoyable, preferred the *Calm* app over the *10% Happier* app, perceived the *Calm* app as more appealing (eg, narrator’s voice and different meditations or background sounds offered), and perceived beneficial effects of meditation on mental health, sleep, fatigue, and pain. Future research is needed to better understand the efficacy of mobile phone meditation on MPN patient outcomes and meditation app design features that enhance uptake among its users.

## Introduction

### Background

Myeloproliferative neoplasms (MPNs) are rare hematological cancers (polycythemia vera, essential thrombocythemia, and myelofibrosis), with a chronic symptom burden that often includes fatigue, sleep disturbances, and depressive and anxiety-related symptoms, to name a few [1,2]. The only potentially curative option is allogenic stem-cell transplantation, but it is reserved for high-risk myelofibrosis patients. Moreover, the current best available pharmacologic therapy does not completely resolve symptoms, and other standard-of-care treatments for MPN are associated with worsened fatigue, inactivity, and a reduced quality of life [3]. Despite this, patients often have a favorable life expectancy (unique compared with most malignancies), with as much as two-third of MPN patients living up to 15 years after diagnosis and some with the same life expectancy as the general population, rendering MPN a c*hronic* cancer condition [4,5]. There is a need to examine complementary approaches in MPNs as a method of self-management of symptom burden for patients.

Mindfulness meditation has gained increasing attention as a complementary therapy for chronic cancer patients, particularly for alleviating a range of symptoms associated with cancer and its treatment (eg, fatigue, emotional distress, and sleep disturbances) [6-8]. Mindfulness meditation is the practice of moment-to-moment awareness, in which the person purposefully focuses on the present moment, without judgement [9,10]. However, there has been minimal research investigating the effects of mindfulness meditation as a complementary therapy in hematological cancer patients, and more specifically, only 1 small feasibility study has been conducted in MPN patients [11,12].

Participating in meditation for anxiety and stress reduction and quality of life in cancer patients is recommended by The Society of Integrative Oncology Clinical Practice Guidelines [13]. However, meditation interventions are often delivered in person (as opposed to home based or remotely), which presents many limitations [14]. In 2017, Gowin et al [15] conducted a survey in MPN patients (n=1676), and 18.97% (318/1676) of the patients reported trying meditation for the management of symptoms, but they said it was burdensome to travel to attend the face-to-face classes. MPN patients face many demands and stressors, feel overwhelmed, are fatigued, are often reluctant to take on commitments for attending and engaging in in-person interventions, and often seek treatment outside of their home city or state (ie, lack of nearby specialized treatment centers) [16,17]. Even when delivery of meditation is virtual or remote, limitations still exist, including the following: (1) there is attendance to a specific weekly schedule, (2) *meditation* programs (ie, mindfulness-based stress reduction) are comprehensive and can be potentially burdensome (90-min sessions, 8-12 weeks long) [18], and (3) traditional mindfulness meditation programs are typically delivered by trained providers, which may be costly and not covered by insurance. There is a need to establish effective modes of delivering mindfulness meditation to chronic cancer patients, such as MPN patients.

Mobile health (the use of mobile wireless technologies for health) [19] has become a topic of considerable interest among cancer patients as a means of promoting self-management of chronic disease for better health outcomes [20]. Most cancer patients own a mobile phone, regularly use mobile apps, and are interested in accessing supportive care information via a mobile app [21,22]. In a recent survey of 1300 cancer patients, 71.00% (923/1300) of the patients reported owning a mobile phone [21]. In a 2018 survey of 631 cancer patients, 74.0% (467/631) of the patients reported regular use of a mobile phone, and 38.9% (246/631) of the patients expressed an interest in supportive care information via mobile apps [22]. In a pilot study conducted by Huberty and colleagues, 96.9% (308/318) of MPN patients indicated that they owned a mobile phone and were willing to download a mobile app to participate in app-based meditation [12]. There are approximately 300 cancer-specific apps available across the major mobile phone platforms (eg, iPhone, Android); however, a majority of available apps have limited evidence to demonstrate their effectiveness and utility, and the evidence of the clinical benefits of commercially available apps for cancer patients is in its infancy [20,23]. Despite minimal evidence in cancer patients, there have been some recent advances in the evidence base, supporting mobile phone-based meditation for health-related outcomes. A recent study conducted by Economides et al [24] investigated the effects of meditation delivered using the Headspace app on stress, affect, and irritability in novice meditators as compared with an active control (ie, audiobook delivered via Headspace app), and they found that participants in the meditation group (n=41) averaged approximately 44 min/week of meditation when asked to complete a total of 10 introductory, 10-min meditations as they desired. Furthermore, participants also saw significant improvements in irritability (Cohen *d*=0.44), affect (Cohen *d*=0.47), and stress (Cohen *d*=0.45) compared with the active control group (n=28). Another recent study conducted by Bostock et al [25] examined the effects of a 45-day (approximately 6.5 weeks) Headspace meditation app intervention on work stress and well-being in healthy workers (n=128) compared with a wait-list control group (n=121), and they found that participants averaged approximately 42 min/week of meditation alongside significant improvements over 45 days in well-being, anxiety symptoms, depressive symptoms, and job strain compared with the control group. Despite the promising findings demonstrated by these aforementioned studies, they were both of relatively short durations (approximately 2 weeks-6.5 weeks), were not powered to determine efficacy, and did not report on features and experiences of users that were most desired for continued participation and engagement with the app. The features, functionality, and experience desired by users of mobile phone-based meditation apps have yet to be thoroughly investigated and reported. A recently published study, Huberty et al [12], investigating the feasibility of mobile phone-based meditation via the *Calm* app and the *10% Happier* app among MPN patients demonstrated the *Calm* app to be more feasible to implement because of higher demand and acceptability. In addition, this study demonstrated limited efficacy, with small effects observed in both apps on anxiety, depression, sleep disturbance, and total symptom burden. This study illuminated some of the aspects of the *Calm* app that may have accounted for the higher demand and acceptability compared with the *10% Happier* app (eg, better app esthetics, more soothing meditation narrator voice); however, more detailed qualitative findings are needed to better understand what features and experience users desire when meditating with a mobile phone app.

### Objectives

Considering the potential benefits of meditation on cancer symptoms, the difficulty for MPN patients to attend in-person meditation interventions, the increasing prevalence of cancer-specific mobile phone apps, and the ease of accessibility to potentially efficacious interventions for symptom management, MPNs are an ideal chronic cancer population with which to gather perceptions and experiences of participation in a consumer-based mobile phone meditation intervention. The aim of this study was to report MPN patients’ (ie, naïve or nearly naïve meditators) perceptions of meditation and explore their experiences in the context of using a mobile phone for meditation after participation in an 8-week, consumer-based meditation app feasibility study. Data presented here will inform the selection of an app for a future efficacy intervention in cancer patients and may provide useful information for content and design features for future meditation apps targeted at cancer patients.

## Methods

### Study Design

Participants were MPN patients who participated in a 4-group, randomized controlled trial, with a cross-over study design to examine the feasibility and limited efficacy of 2 different consumer-based meditation mobile phone apps in MPN patients: *Calm* and *10% Happier*. We used the *Calm* app for this study, as *Calm* is one of the most popular consumer-based mobile apps (ie, Apple’s app of the year in 2017), the team developed a relationship with *Calm* to conduct research using the app, and *Calm* agreed to provide the memberships to the app and share the tracking data with the research team, without cost. The *10% Happier* app was chosen, as the app was one of the competing meditation mobile apps of *Calm,* and *10% Happier* also agreed to provide the memberships to the app and share the tracking data with the research team, without cost. Both mobile phone apps are available across all major mobile phone platforms (ie, Android and iOS). Both the *Calm* app and *10% Happier* app were developed for the general population and not necessarily for a particular chronically ill population. In addition, both of these apps have a free option with limited accessibility, as well as a paid option with full accessibility. The findings and a detailed overview of the methods from the feasibility study are reported elsewhere in the parent paper [12] (ClinicalTrials.gov, NCT03726944). Briefly, participants were randomly assigned to 1 of 4 different groups that each comprised 2 different conditions lasting 4 weeks each. Group #1 received the *10% Happier* app, followed by the *Calm* app; Group #2 received the *Calm* app, followed by the *10% Happier* app; Group #3 received an educational control condition, followed by the *10% Happier* app, and Group #4 received an educational control condition, followed by the *Calm* app. When participating in a meditation app condition, participants were asked to meditate for 10 min/day on each day of the week. Those in the educational control condition received a handout describing evidence-based fatigue management strategies. All participants had autonomy to use the app as they desired after completing the prescribed daily meditation. Each app housed a library of meditations and content from which participants could choose. Emails were sent to all participants at the beginning of each week to remind them to meditate (ie, use the app). The qualitative portion of the study was designed to explore the perceptions of MPN patients practicing meditation either for the first time or as fairly inexperienced users and to explore their experiences in the context of mobile phone delivery.

### Recruitment

MPN patients (n=128) were recruited on the Web through MPN organizational partners, with a flier outlining the study and its requirements. The study was advertised as a mobile phone app meditation study. MPN patients interested in the study were asked to complete a Web-based eligibility questionnaire, administered via Qualtrics (Provo, UT), and if eligible, a phone call was arranged by the staff to complete informed consent, followed by electronic signature. Patients were eligible if they (1) had a diagnosis of essential thrombocythemia, polycythemia vera, or myelofibrosis, (2) owned a mobile phone and were willing to download and use a meditation app, (3) could read and understand English, (4) were aged 18 years or older, (5) were willing to be randomized to 1 of 4 different groups, (6) were not regular meditators (ie, engaged in <10 min/day of meditation on <5 days/week for the past 6 months), (7) were not regularly engaged in tai chi, qigong, or yoga (ie, engaged in >60 min/week each week), (8) were neither currently using the *Calm* app nor the *10% Happier* app, and (10) were currently residing in the United States.

#### The Calm App

**Figure 1:**
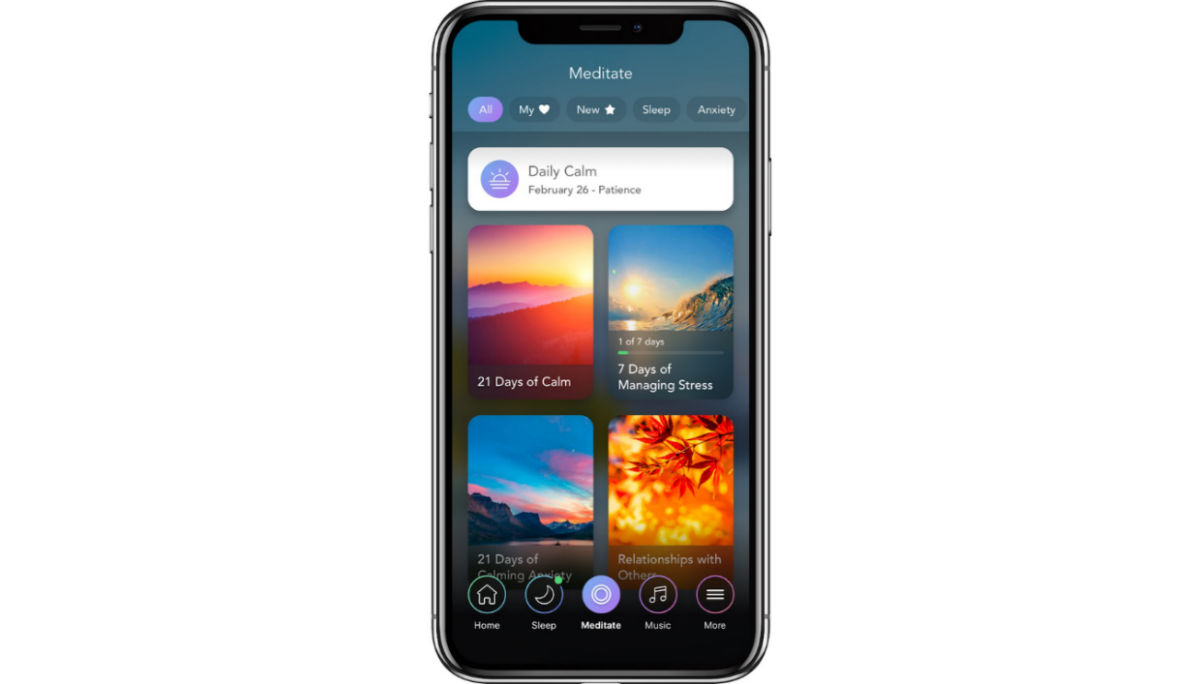
The *Calm* app.

The *Calm* app was downloaded onto the participant’s mobile phone, and the app was available to those with an iPhone or an Android phone. The *Calm* app’s introduction to meditation incorporated basic, educational information for those new to meditation, while introducing brief experiential practices. Daily meditations were called the Daily *Calm,* and these were new and unique, provided by the app each day. The daily meditations had a different focus (eg, practicing patience, loving, kindness, and gratitude), and these were approximately 10 to 12 min in length. Meditations were also selected from a library of meditations with the app. In addition, the *Calm* app also offered other features, such as Breathe Bubble, Sleep Stories, *Calm* Body, *Calm* Music, *Calm* Masterclass, and Background Scenes. *Calm* provided researchers with the usage data for each study participant (ie, number of minutes; see Figure 1).

#### The 10% Happier App

**Figure 2:**
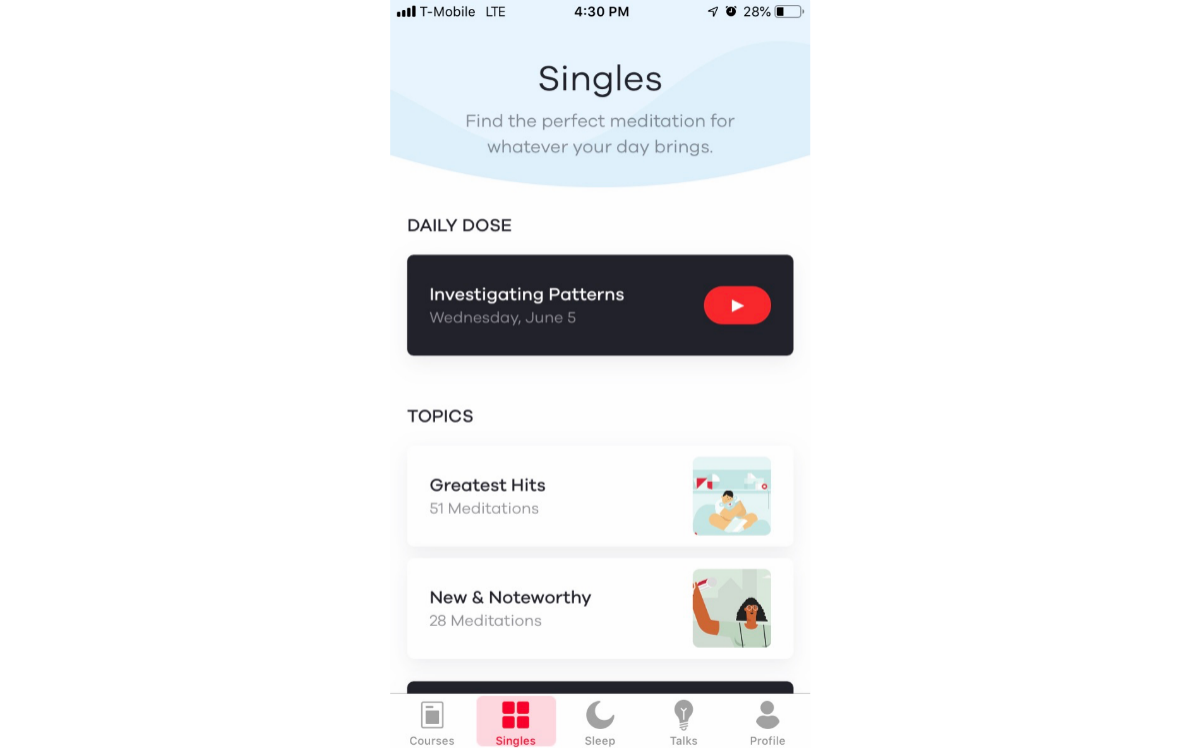
The *10% Happier* app.

The *10% Happier* app was downloaded onto the participant’s mobile phone, and the app was available to those with an iPhone or an Android phone. The *10% Happier* app’s introduction to meditation incorporated basic information for those new to meditation. Daily meditations were selected from a library of meditations included within the app. Each of the meditations had a different focus (eg, grief, gratitude, choice, and letting go), and these were approximately 10 to 12 min in length. *10% Happier* primarily offers individual, guided meditations and short courses (eg, Meditation for Skeptics, Phrases for Stress). *10% Happier* provided researchers with the usage data for each study participant (ie, number of minutes; see Figure 2).

### Qualitative Interview Procedures

At the end of the 8-week feasibility study [12], participants were given an option to participate in a 15-20-min telephone interview, conducted by trained student research assistants. Before conducting each interview, the research assistants explained the purpose, the amount of time the interview would take, and the voluntary nature of participation (ie, the participant’s ability to skip questions or end the interview at any time). The interview questions, as shown in Table 1, were developed by the research team to determine perceptions and explore experiences in the context of mobile phone delivery of meditation, as well as provide further insights into how such interventions might be beneficial for the target population.

A semistructured interview script was used for each interview, including open-ended questions and additional probe questions to garner further information from the study participants. Although some probe questions were predefined (see Table 1), interviewers were also free to ask additional questions for clarification or expansion of the specific responses provided by participants. The relatively short and semistructured approach to qualitative data collection allowed for the interviews to remain focused while being open and flexible for participants to describe their experiences [26,27] of using the meditation apps from their personal perspectives and in their own words. With the permission of participants, all interviews were audio recorded for transcription.

All interview transcripts were imported into NVivo 12 (QSR International) for coding and analysis, using a combination of deductive and inductive methods to organize the data, generate categories, and develop themes and subthemes [28,29]. Braun et al [26] describe thematic analysis as “a method for identifying, analyzing, and reporting patterns (themes) within data” (page 6). It is not bound to any particular theoretical framework, and it can therefore be used flexibly within a range of different epistemological and ontological perspectives. The hybrid deductive and inductive approach to thematic analysis used in this study reflects the thematic analysis approach described by Braun et al [26] and Swain et al [27], in which both the existing knowledge and understanding of the researcher, as well as the semantic content of the research data, are involved in developing codes to represent particular constructs. We used a semistructured approach to data collection, in which the raw interview data naturally fell into a number of higher-level themes related to the predefined questions or structured aspects of the interviews. Within these higher-level themes, inductive thematic analysis methods were used to identify relevant lower-level codes or constructs from the transcribed raw interview data, with chunks of data being assigned to codes and labeled to reflect their meaning [26]. The overall coding process, which was carried out by a highly experienced qualitative research specialist and member of the research team, involved an iterative process, with several stages in which codes and their corresponding labels were reviewed, revised, and in many cases, grouped or categorized within intermediate-level codes or themes. This continued until the overall distribution and definition of themes and subthemes were felt to most accurately reflect the body of research data and the reported experiences of the participants. The findings of the study are reported by key themes below and illustrated by verbatim quotes from the interviews to convey the real-life experiences of the participants. Quantitative counts of the numbers of participants reporting particular types of views on or experiences of the meditation intervention are included in tables and the narrative in a summative style of analysis.

**Table 1:** Interview questions.

Number	Question
1	Which meditation app or apps did you use during your participation in this study?
2	If both Calm App and 10% Happier App were used, which meditation app did you prefer or enjoy more and why?
3	What did you like most about using this app or these meditation apps?
4	What did you like least about using this app or these meditation apps?
5	How do you feel that participating in meditation has impacted you?
Probe	Fatigue levels? Sleep quality? Self-esteem? Mental well-being? Overall health?
6	Have you noticed any other changes in your life that might be associated with your participation in meditation?
7	Before starting this study, how did you feel about meditation?
Probe	Had you ever participated in meditation before?
8	Now that you have completed this study, how do you feel about meditation?
Probe	Do you think you will continue to meditate moving forward? If yes, why do you think you will continue meditating moving forward? If no, is there anything that would encourage you to continue meditating? Are there any other complementary approaches that you are interested in trying? (Tai Chi? Qi Gong? Massage?)
9	If another MPN^a^ patient asked you what you thought about him or her participating in meditation, what advice would you give?

^a^MPN: myeloproliferative neoplasm.

### Approval and Consent

This study was approved by the Institutional Review Board at Arizona State University, and all participants signed an informed consent before participating in the study.

## Results

### Characteristics of Study Participants

The total sample included 48 MPN patients. Participants were aged 59 (SD 10) years, had a body mass index of 27 (SD 6) kg/m^2^, and were primarily female (73%; 35/48) and Caucasian (94%; 45/48). In addition, the majority of participants had an annual income >US $61,000 (60%; 29/48), had a Bachelor’s degree or higher (69%; 33/48), and were married (81%; 39/48). The most common MPN diagnosis was polycythemia vera (42%; 20/48), followed by essential thrombocythemia (33%; 16/48) and myelofibrosis (25%; 12/48). Most were diagnosed >3 years ago (58%; 28/48). Across demographic variables, most were comparable to what is typically seen in MPN patients, with the exception of the majority being female (approximately 50%-55% female is the typical proportion in MPNs) [1]. Fatigue at baseline averaged approximately 5.9 (on a scale of 0-10; 0=absent; 10=worst imaginable) and total symptom burden at baseline, as measured with the validated MPN Symptom Assessment Form Total Symptom Score (MPN-SAF TSS), averaged approximately 39.4 (on scale of 0-100; higher score indicating greater symptom burden). These baseline scores were slightly higher than what is typically seen in MPN patients (mean fatigue score is approximately 4.0-4.5, and mean MPN-SAF TSS is approximately 20.0-25.0) [1]. Upon completion of the study, weekly meditation participation averaged approximately 99 min/week for those who participated in the *Calm* app and approximately 36 min/week for those who participated in the *10% Happier* app. A total of 68% (22/32) and 20% (7/36) of *Calm* and *10% Happier* participants, respectively, averaged >70 min/week of meditation (70 min/week was prescribed). A total of 22 participants used both the *Calm* and *10% Happier* apps, 10 participants only used the *Calm* app, and 14 participants only used the *10% Happier* app. Note that not all participants were asked or did not provide an answer to the same questions. Therefore, total frequencies (n=46) do not equate to total sample size (n=48) of this study.

### Experiences and Perceptions of Meditation Before the Study

A majority of participants (60%; 29/48) reported some previous experience with meditation before participating in this study, although as a cutoff for inclusion in the study, participants could not have meditated more than 10 min/day on more than 5 days/week in the previous 6 months [12]. However, despite the high proportion of patients who had some experience with meditation in the past, less than half of the qualitative study participants (44%; 21/48) indicated that they were open minded about meditation (ie, willing to learn about meditation). Although some participants indicated that they were willing to learn about whether meditation might be beneficial for them personally, others reported that they had never been interested or had never given meditation much thought:

I had no idea how it was going to affect me, or...whether I was going to like it or not, whether I was going to be able to do it.

In fact, 19% of the sample (9/48) indicated that they had negative preconceptions or concerns about participating in meditation, such as the belief that they would not be able to clear their mind effectively or that the sessions would be long and tedious. Some held the unrealistic idea that the goal of meditation is to sit cross legged like a Buddhist monk or to achieve a completely blank mind:

I (had) that preconceived thing of, you know, people sitting there with their legs crossed humming.

The idea of turning, kind of turning the world off or my mind off for a block of time seemed really unrealistic.

Other types of concerns reported by some of the participants are related to the fear that others (egg, peers, friends, and family) would find them weird for meditating or that they would be vulnerable to subliminal messages in the meditation recordings:

I have heard of meditation classes...you would sit and listen to someone talk or describe breathing and it would be half hour or an hour and I thought that was kind of a long span to kind of sit there and just mellow out.

(I) always worry about...subliminal messages in meditation. Because you’re opening your mind so much...I always feel cautious and I am guarded.

### Perceptions of Meditation After the Study

After participating in the study, a majority of participants expressed the view that they had enjoyed using the meditation apps, with a considerable number (17/48) reporting that their perceptions of meditation had improved or become more positive as a result of the study. Their responses indicated that there were 2 main reasons for becoming more positive about meditation as a result of participating in the study. First, some of the participants indicated that the practice of meditation had proved to be easier than they had expected and that it was not necessary to totally clear the mind during meditation or allocate a lot of time to the practice:

If your mind starts thinking about things that’s totally okay...that’s totally normal...don’t expect to be perfect, I mean it was just so much reassurance about how much of a learning curve is involved.

It doesn‘t have to be a chunk of time, it can just be a few minutes here or there and that’s what for me really clicked and made me realize, I don’t have to block off a chunk of time, it can be bits of time here and there...and that for me made it seem more realistic and reasonable.

Others indicated that their stereotypical ideas about meditation had been broken down, as they realized that it is a practice that everyone can benefit from:

I didn’t picture it like a cult, but when you think of meditation, that’s kind of what you think of. And it wasn’t, at all...You know, it’s just a way to calm yourself down, which I think is very beneficial. So, it was nice, I mean, it’s pretty much something anybody can do.

It was very simplistic and very easy and it didn’t have anything to do with - you know - some of this some of this other stuff...the gurus I guess...some of the meditation things that you’d see on TV.

Some participants indicated that they felt more positive about meditation as a result of experiencing direct benefits from it, such as feeling more calm or relaxed or being able to manage their pain better:

I never believed in meditation, but in medication, and now it’s different...It does work.

It was kind of nice to...be able to just relax and kind of let go.

A few participants also commented that they had enjoyed the flexibility of being able to meditate anywhere, including alone at home, without having to go to a class:

I had (a) procedure three hours away in Portland...So that was cool that I was able to take it with me.

I don’t get to get out very much... the thing I like the best about this is that I can just do it at home ...

As a result of these improved perceptions of meditation, a majority of the study participants (41/48) indicated that they intended or were at least considering continuing their meditation practice after the end of the study or were already doing so. More than half of all participants (29/48) reported that they planned to continue meditating with one of the study apps at least for the remainder of the free trial period, and some were considering paid subscription options for when this came to an end. Others (16/48) indicated that they do plan to continue meditating, but they planned on continuing by using different apps, meditating independently without the use of an app, or attending a class. For some, participating in the study had either given them a new or renewed interest in meditation or provided them with methods through which they planned to continue using without the app itself. A range of reasons were given for continuing meditation, such as experiencing the benefits, being able to meditate even when going through periods of severe illness, managing pain, or helping them sleep. A participant wanted to continue in preparation for future times when the participant’s condition might worsen and the participant would use meditation to help manage symptoms. Only 5 participants indicated that they were not likely to continue using the apps or meditating at all. All participants indicated that they would recommend that MPN patients should at least try mobile phone meditation. A participant expressed the view that it is important for MPN patients to exert an element of control over the sense of worry and lack of control that comes with having an MPN and that meditation practice could help provide this. Others felt that meditation was worth a try, as it was not very difficult to do and had the potential to calm emotions, ease day-to-day concerns, relieve stress, or improve sleep:

The idea of totally emptying your mind relieved a lot of worries so it’s worth trying.

There’s a constant low-grade anxiety that comes along with it (MPN), and honestly the meditation helps with that a lot.

For anybody who does have fatigue, I think it will help you settle yourself down and maybe improve the quality of sleep. So...yeah so, I would say go for it.

### Perceptions of the Calm and 10% Happier Apps

#### Overall Perceptions of the Apps

In general, mobile phone-guided meditation was well accepted and liked among the participants, and factors, such as the length of the meditations and the instructional content, seemed to contribute to the participants’ enjoyment of both apps. Most participants who expressed a view on the issue agreed that the level of instruction in the apps was suitable for beginners:

I had never done meditation...I felt she really did a good job of teaching me how to do it and it didn’t take me long to catch on.

Calm

I liked what they call the basics, the, the sixteen-course introduction to meditation...I thought those were very informative and helpful.

10% Happier

With regard to length of the meditations, only 10% (5/48) of the sample commented that they would have preferred the length to be different (ie, longer or shorter). It seemed that most participants felt that a commitment of 10 min of meditation was not too big of a time commitment and long enough to feel accomplished when done, but they felt that it was not so long that they became impatient for the meditation to end:

Ten minutes wasn’t a big amount of time commitment and it seemed really adequate and there was a feeling of success.

I think the ten-minute ones are really good. It’s enough to kind of settle yourself down, and kind of get into it, but yet, you’re not thinking ‘oh my gosh, how much longer is this going on.’

Of those who were more dissatisfied with the length of meditations, some mentioned that they would have preferred shorter sessions at first, whereas others would have liked to see a steady progression in length throughout the study:

When you first start out...I think they need to be shorter...because your brain, isn’t wrapped around what you’re doing...so, you know, I would find myself losing focus, really quick.

I think I would have liked them to...every week maybe add a few minutes...so that we went from maybe ten to maybe twenty.

A total of 7 of the participants who used both apps expressed their views, indicating that they liked both apps equally or felt that they complemented each other well; therefore, they indicated not preferring a single app. Many of their comments suggested that the *10% Happier* app was seen by them as providing a better introduction to meditation, whereas *Calm* built on this with more effective meditation techniques.

#### Perceptions of the Calm App

Of those participants who expressed a preference for one app over the other, a majority (91%; 20/22) expressed a preference for the *Calm* app. The main reasons for participants preferring the *Calm* app included the soothing nature of the narrator’s voice, the calming background sounds on the app, and its overall appealing nature and layout:

It was very relaxing. In fact, like, it would put me to sleep in five minutes.

I set it to do ocean sounds during the meditation. Which I think really helped me as opposed to the other one was just - kind of like dead air.

Not only do I like the things that they’re saying but...the pictures that they have...you can just get lost in those things [laughs]...it totally sets you up to be calm.

Several reported that they especially liked the Sleep Stories or other stories included in the *Calm* app, and they found them to be meaningful or relaxing:

The stories at the end...some of them really touched me a lot and I kind of want to go back and hear them again because I want to keep...the focus of what things are all about in my mind. So as the day goes on I can remember what’s really important.

Participants who preferred the *Calm* app also referred to the wide range of meditations and the options available to pick and choose these or to tailor the app to their own preferences:

I was...really impressed...by how many choices and different types of anxiety or sleep or whatever your choice would be, there’s things available and...different backgrounds and different music...

I liked that I could customize for the background noise and for the wallpaper

#### Perceptions of the 10% Happier App

The minority (27%; 6/22) of those who had used both apps expressed a preference for *10% Happier* over *Calm*. Of these, some indicated that they had preferred the personal style of the narrator and found they could relate to his experience, whereas others liked the intellectual content and tone of this app and felt they had learned a lot about meditation from the app. A total of 3 participants who had used *Calm* first and then the *10% Happier* app said that the initial meditation videos within the *10% Happier* app would have provided a better introduction to meditation. Among the participants as a whole, including those who had only used *10% Happier* and those who had used both apps, the types of features liked about *10% Happier* included the way in which it provided a good introduction to meditation for nonexperienced meditators: the relatability of the narrator’s style, the informative, educational style of the app, and the wide range of meditation topics available for selection. Overall, 12 participants made positive comments about the introductory videos included in *10% Happier,* indicating that they were interesting and a good lead into the meditations. However, some noted that they skipped these introductory videos, as they were felt to be unnecessary and even tedious once the participants were familiar with the meditation techniques:

They were good at first, but then it was really kind of dull. It was like, alright, guys, I don’t need this, let’s move on.

Some participants indicated that they disliked some of the narrative voices used in *10% Happier* and that they were “put off” by the narrator’s personality or “put off” by the overall style of the narratives, which they found too impersonal:

I had a hard time getting into him...it was just the...the way he talked...it just wasn’t right for me.

They used the word “the” instead of “your”, and I feel that meditation is a real personal sort of a thing...Calm would address me personally, even though it’s an electronic device. But...the other one would use “the”, it would seem very impersonal. So, I just didn’t connect with it.

Others disliked the fact that there were no background sounds in between segments of the narrative, and they preferred the calming sound effects used in the *Calm* app:

I kept sitting there waiting for something else...you weren’t sure when he was going to stop talking and start talking again.

It was complete silence and I didn’t know if I’d been disconnected or if I’d fallen asleep or if it was over.

The main likes and dislikes of the *10% Happier* app are shown in Table 2 by numbers of participants citing each type of factor.

**Table 2:** Views on the *Calm* and *10% Happier* apps by number of participants citing each type of factor.

Factor by category		*Calm*, n	*10% Happier*, n
**Main likes**			
	Introductory videos	—^a^	6
	Range of topics or background sounds	11	4
	Overall format and ease of use	8	0
	Effectiveness in promoting meditative state	8	0
	Narrator’s voice or personal narrative	6	6
	Effectiveness/quality of information	5	5
	Stories	5	—
	Length of meditations	4	0
**Main dislikes**			
	Technical/navigation features	5	4
	Background sounds/soundtracks	4	10
	Too much talking/overall style	2	6
	Style or limited variety of narration	2	6
	Content of meditations	2	2

^a^Not applicable.

### Perceived Impacts of Using the Meditation Apps

A total of 25% (12/48) of participants reported no perceived benefits of meditation on their health. However, a majority indicated that use of the meditation apps had a positive impact on various aspects of their mental or physical well-being. Improvements in mental health were the most commonly cited benefits of using the apps. More than half of the sample (26/48) reported a positive impact on their ability to manage stress or anxiety, 13 participants indicated that meditation helped them feel calmer or more focused, and 8 participants reported an improved sense of general well-being since participating in the meditation study. Those who reported an impact on their ability to manage stress or anxiety highlighted ways in which the tools or strategies they had learned from the apps, such as focusing on breathing, had helped them in stressful situations or the ways in which meditation had helped them deal with worries about their condition:

I got some stuff going on at work that’s really frustrating and there were times at work that I would just sit down and close my eyes and concentrate on my breathing.

I had to have an MRI and I completely used the meditation while I was in the MRI and it worked. And it helped me, it was amazing.

Others discussed ways in which they were feeling calmer or more focused in their lives as a result of the meditation practice, or they referred to a general improved sense of mental well-being:

I think I learned a little bit about...focusing on getting interfering thoughts out of my head

It just leads to being a lot more focused...And a lot more attentive I think with everything.

I think it puts me in a better frame of mind.

In addition, 44% (21/48) of the participants reported improvements in sleep, resulting from the meditation practice, including an improvement in sleep quality, as well as falling asleep more quickly. Some mentioned the sleep-focused meditations and stories on the *Calm* apps as being particularly helpful for better sleep:

Once I started meditating, it seemed to make a real shift there so that I would be able to go to back to bed and go back to sleep. And, and so I would wake up more refreshed in the morning.

The sleep stories were especially good...Yeah, they really do help me just focus on something, I guess, and helped me go to sleep.

Many of those who said they were falling asleep faster were deliberately using their meditation practice at bedtime for this reason, whether or not they usually had problems falling asleep:

I was using it to help me fall asleep because at night time I, I find that I don’t sleep well, I can’t fall asleep, so I found it very soothing and comforting.

**Table 3:** Number of participants reporting positive impacts.

Factor	n
Managing stress or anxiety better	26
Easier to fall asleep	21
Better quality of sleep	21
Feeling calmer or more focused	13
Reduced fatigue	11
Improved sense of well-being in general	8
Ability to manage pain	4

Although 9 participants reported no impact on quality of sleep, in some cases, this was because of the fact they were not experiencing sleep problems when they began. Similarly, although not all participants were experiencing fatigue as one of their symptoms, 23% (11/48) of the participants reported improvements in fatigue as a result of meditation. For some, this was because of the fact that meditation helped them sleep better so that they were less tired during daytime, whereas others felt that the meditation schedule just enabled them to take a rest break, which helped recharge their energy.

I’ve had more energy too, and again, you know it’s that because I’m sleeping better.

I think there are times when I thought “Oh I could lay down, I could take a nap” and I would just lay down and listen to a meditation and you know feel more relaxed and that eased and then get up and function, you know?...I think it gave me that break where ok you can take time out of your day and do a little meditation and then maybe you feel like you can get up and do things.

Finally, 8% (4/48) of the participants reported that meditation had been helpful in reducing or helping them manage pain.

I have noticed if I started meditating and had a headache, the headache seemed to go away.

Some participants who reported perceived impacts of meditation on various aspects of their physical or mental well-being were asked how long it had taken after starting the meditation program for the effects to become apparent to them. Of the 10 participants who responded, most indicated that it had been about 1 to 2 weeks before noticing changes, whereas a few had either noticed effects very quickly or after a longer period of about 3 weeks. Table 3 shows the number of participants reporting various types of positive impacts from use of the meditation apps.

## Discussion

### General Discussion

The aim of this study was to report MPN patients’ (ie, naïve or nearly naïve meditators) perceptions of meditation and explore their experiences in the context of using a mobile phone for meditation after participation in an 8-week, consumer-based meditation app feasibility study. The qualitative data provide in-depth information that can be used (combined with the results of our published feasibility study) [12] in the selection of an app for a future efficacy intervention in cancer patients. The information may also inform content and features for future meditation apps targeted at cancer patients.

Overall, the qualitative findings of this study indicate that mobile phone-guided meditation was very well accepted and liked among MPN patients, although most patients who experienced both expressed a greater liking for the *Calm* app over the *10% Happier* app. However, regardless of which app patients preferred, they felt that mobile phone meditation positively impacted physical and mental well-being, including fatigue, sleep quality, and their ability to manage stress and anxiety. Overwhelmingly, all patients would recommend meditation to other MPN patients; most reported likeliness of continuing to meditate, and a majority reported a likeliness to continue meditating with one of the consumer-based apps. This was despite the finding that more than half of the participants had negative preconceptions about meditation, partially because of the stigma surrounding it [30]. Therefore, future studies are warranted to develop and test the introduction and educational components of meditation apps to assure they are tailored for the specific populations’ preconceptions about meditation.

After participating in the study, a majority of participants indicated that they had enjoyed using the meditation apps. As mentioned above, most expressed the view that around 10 min was a suitable length of time for meditation sessions, and this seemed to contribute to their enjoyment of the apps. There is little previous literature available to suggest the ideal dose or length of meditation, as studies have varied quite considerably in their prescription [31]. This is especially the case in hematological cancers, with no research being conducted to date in MPN patients, before our recent feasibility study [11,12]. Meditation interventions have shown effects with time spent in meditation ranging from 10 min to 2 hours and from 1 day a week to a daily practice [32-34]. In a feasibility study of 5-, 15-, and 30-min meditation sessions, the 15-min sessions were the most feasible to implement among health care professionals [35]. This falls in line with current practical recommendations, in which it is recommended that beginners begin by meditating between 10 and 30 min per session [36]. Therefore, it seems that consumer-based apps that offer 10-min daily meditations are in line with what is feasible and most practical, on the basis of the current literature, and this likely contributed to the participants’ enjoyment of the length of meditations. However, some of the participants in this study expressed a preference for different or increasing lengths of meditation, which suggests that both the length of meditations at the outset of an intervention and the meditation’s extension over time might usefully be explored in future feasibility studies. In a recent study by Huberty et al [17] that investigated the feasibility of 12 weeks of Web-based, home-based yoga in MPN patients, participants who completed the intervention noted that the flexibility and convenience of being able to do yoga at home instead of going to a studio was one of the best features of a remote intervention. Cancer patients report barriers that make it difficult to participate in in-person interventions [16], and mobile phone-based meditation helps in addressing these issues, as participants can participate in meditation when they want and where they want. It is likely that the convenience and flexibility of mobile phone-based meditation contributed to its feasibility, with some participants in this study commenting on the flexibility of this approach, as noted earlier.

Of those participants who reported a preference for one app over the other, a majority (91%; 20/22) expressed a preference for the *Calm* app. There may be other unique features of the *Calm* app that made it more preferable compared with the *10% Happier* app. Despite a lack of research on meditation apps, there has been some research investigating the desired features of physical activity–based and, more broadly, health behavior change–based apps. This research suggests automatic tracking and ease of tracking activity and progress, as well as integrated features (eg, syncing with social media platforms and music apps), are desired and make apps more likely to be used [37,38]. Although both the *Calm* app and the *10% Happier* app track meditation progress for the users, the *Calm* app immediately displays the current streak of meditation days and offers the users to (1) share the daily quote on social media (ie, Instagram, Facebook) or via text, (2) see their profile of meditation statistics and progress (ie, number of meditations completed, longest streak of meditations completed, and total meditation min) and share with others (ie, social media, text, and email), (3) rate the session, and (4) give 30 days of *Calm*. In comparison, the *10% Happier* app displays the days of the last week spent in meditation, and if the users choose, they can also see the minutes of meditation and number of sessions, and then they can share their progress.

There is also little research investigating the features of meditation apps that are most desired or wanted by users. This is despite the over 300 mindfulness- or meditation-based mobile phone apps available across the Google Play Store and Apple’s App Store [20,23]. Laurie et al [39] suggest that meditation apps should be designed so that (1) users can be encouraged to find a proper time and place to meditate regularly and (2) users can be encouraged to integrate the mobile phone app with other features on their phone, such as alarms and calendar notifications reminding them to meditate; in addition, meditation apps (3) offer users alternatives to meditating sitting still in the case that the user wants to engage in movement of some sort as well (eg, mindful movement, such as yoga or tai chi), and (4) the app itself is more interactive and less passive. There is still much to be learned about the use of mobile apps to deliver meditation and the way to cater the app to the individual. As discussed above, most of the participants in this study reported mental or physical health benefits. This is not surprising, as mindfulness meditation has been shown to improve a range of cancer-related symptoms, including treatment fatigue, emotional distress, and sleep disturbances, to name a few [6-8]. Meditation may have improved mental health (eg, anxiety- and stress-related symptoms) through its calming effects on the autonomic nervous system and its ability to help improve attentional control and regulation of emotions [40-42]. Some of the other reported benefits by participants, including sleep and fatigue in particular, may also be related to one another, as some reported that they felt less fatigued, as they had better quality sleep. It is possible that meditation helped participants sleep better because of a reduction in sleep-interfering ruminating cognitive processes [6,8,43]. The improved sleep may have then led to participants feeling less tired throughout the following day. However, it is also possible that meditation targeted fatigue indirectly through another mechanism. Meditation has been shown to reduce inflammation, and inflammation is a key driver of MPN fatigue [44-46]. Participants who used the *Calm* app mentioned the use of both the Sleep Stories and the background music nature sounds to help them fall asleep. Sleep Stories on the *Calm* app comprise a narrator telling a story or ancient fable in a bedtime story format. The Sleep Stories are intended to be relaxing and soothing, thereby helping listeners fall asleep. The background nature sounds available on the *Calm* app range in style, from the sounds of running water and birds chirping to the sound of rain on leaves and thunder rumbling in the background. Research has demonstrated the beneficial effects of listening to nature sounds or music on the ability to fall asleep and on sleep quality in both cancer and noncancer populations [47-49]. In regard to the Sleep Stories helping participants fall asleep, there is no research specifically investigating the effects of Sleep Stories and their impact on sleep outcomes. However, there has been research conducted using bedroom story routines that have been associated with better sleep outcomes (eg, improved sleep quantity and quality) [50]. In this study, it is unknown whether study participants listened to the Sleep Stories as part of a nightly routine or on an as needed basis to help them fall asleep. Research is needed that examines the effects of *Calm’s* Sleep Stories and whether stories by themselves help improve sleep outcomes or whether stories integrated into a nightly routine is more efficacious for sleep outcomes. In this study, exactly half (24/48) of the participants indicated that they had stuck to a regular meditation schedule over the duration of the study. However, the time of day in which participants chose to meditate varied. Most (14/24) indicated that they meditated at night, before going to sleep. Conversely, the other 10 reported that they preferred to meditate at any other time of the day, including the morning, afternoon, or early evening. In the study by Laurie et al [39] mentioned earlier, the *Headspace* users (n=16) also had varied patterns of use. It could be that most participants in this study meditated at night, as it was a way of helping them fall asleep. It does seem that having a routine-oriented approach to daily meditation is important, as participants in the *Headspace* study indicated that the toughest part of meditating regularly was identifying a routine time, sticking with that time, and then integrating that routine into their busy lifestyles. The authors [39] suggested that the design of meditation apps should have content that teaches users how to fit meditation into their lifestyle. It may be that meditating at a specific time each day is associated with a higher likelihood of consistent meditation, but future research in this area is warranted to explore this further.

### Limitations

This study is not without limitations. First, participants recruited for this study were not blinded to the nature of the intervention being tested. Participants knew they were volunteering for a meditation intervention delivered via a mobile app. It is possible that this could have attracted interested participants who were more likely to find using the meditation apps enjoyable and likeable. Second, qualitative findings are from a convenience sample of participants who agreed to participate in a postintervention interview, derived from the larger sample that completed the study as a whole. The interview was not required of all study participants, and it is possible that this led to a biased sample of interviewees. In addition, as *Calm* and *10% Happier* offer other content besides meditation (ie, sleep stories, interviews with experts, and educational classes and courses) and as participants had autonomy to use other content on the app, it is possible that participants could have had slightly different interventions when compared with each other. However, this was also built into the nature of the feasibility trial, and this partially encouraged participants to explore other features of the app. Future efficacy studies should aim to deliver a stricter intervention.

### Conclusions

On the basis of the findings of this study, more research is needed to better understand the effects of mobile phone meditation on MPN patients and, more broadly, on cancer patients as a whole. The qualitative findings of this study suggest that MPN patients enjoy mobile phone meditation and experience beneficial effects on their mental health, sleep, fatigue, and pain from using a meditation app, but they prefer the *Calm* app (as compared with the *10% Happier* app). In addition, patients identify with certain design features that make a mobile-based meditation app more appealing (eg, soothing sounds and backgrounds, integration with social media platforms, automatic tracking of progress, and user statistics). Future research is needed that investigates the efficacy of mobile phone-based meditation and further explores the optimization of meditation app design and features to enhance uptake among users. Furthermore, researchers should explore the specific types of meditation sessions and the specific features of the apps that were accessed to better guide recommendations for its users.

## Abbreviations

MPN: myeloproliferative neoplasm
MPN-SAF TSS: myeloproliferative neoplasm Symptom Assessment Form Total Symptom Score
